# Rare Variants in Calcium Homeostasis Modulator *1* (CALHM1) Found in Early Onset Alzheimer’s Disease Patients Alter Calcium Homeostasis

**DOI:** 10.1371/journal.pone.0074203

**Published:** 2013-09-17

**Authors:** Fanny Rubio-Moscardo, Núria Setó-Salvia, Marta Pera, Mònica Bosch-Morató, Cristina Plata, Olivia Belbin, Gemma Gené, Oriol Dols-Icardo, Martin Ingelsson, Seppo Helisalmi, Hilkka Soininen, Mikko Hiltunen, Vilmantas Giedraitis, Lars Lannfelt, Ana Frank, MªJesús Bullido, Onofre Combarros, Pascual Sánchez-Juan, Mercè Boada, Lluís Tárraga, Pau Pastor, Jordi Pérez-Tur, Miquel Baquero, José L. Molinuevo, Raquel Sánchez-Valle, Pablo Fuentes-Prior, Juan Fortea, Rafael Blesa, Francisco J. Muñoz, Alberto Lleó, Miguel A. Valverde, Jordi Clarimón

**Affiliations:** 1 Laboratory of Molecular Physiology and Channelopathies, Department of Experimental and Health Sciences, University Pompeu Fabra, Barcelona, Spain; 2 Memory Unit, Neurology Department, Hospital de Sant Pau (Sant Pau Biomedical Research Institute), Universitat Autònoma de Barcelona, Barcelona, Spain; 3 CIBERNED, Center for Networked Biomedical Research into Neurodegenerative Diseases, Madrid, Spain; 4 Department of Public Health, Geriatrics, Rudbeck Laboratory, Uppsala University, Uppsala, Sweden; 5 Institute of Clinical Medicine – Neurology, University of Eastern Finland and the Department of Neurology, Kuopio University Hospital, Kuopio, Finland; 6 Institute of Sanitary Research “Hospital la Paz” (IdIPaz), Madrid, Spain; 7 Centro de Biología Molecular Severo Ochoa (CSIC-UAM), Madrid, Spain; 8 Neurology Service, University Hospital Marqués de Valdecilla (University of Cantabria and IFIMAV), Santander, Spain; 9 Alzheimer Research Center and Memory Clinic, Fundació ACE, Institut Català de Neurociències Aplicades, Barcelona, Spain; 10 Hospital Universitari Vall d’Hebron, Institut de Recerca, Universitat Autònoma de Barcelona (VHIR-UAB), Barcelona, Spain; 11 Neurogenetics Laboratory, Division of Neurociences, Center for Applied Medical Research (CIMA) University of Navarra Medical School, Pamplona, Spain; 12 Institut de Biomedicina de Valencia-CSIC, Valencia, Spain; 13 Neurology Department, Hospital Universitario “La Fe”, Valencia, Spain; 14 Neurology Department, Hospital Clínic, IDIBAPS, Barcelona, Spain; 15 Molecular Basis of Diseases Unit, IIB-Sant Pau, Hospital de la Santa Creu i Sant, Pau, Barcelona, Spain; School of Medicine and Health Sciences, University of North Dakota, United States of America

## Abstract

Calcium signaling in the brain is fundamental to the learning and memory process and there is evidence to suggest that its dysfunction is involved in the pathological pathways underlying Alzheimer’s disease (AD). Recently, the calcium hypothesis of AD has received support with the identification of the non-selective Ca^2+^-permeable channel CALHM1. A genetic polymorphism (p. P86L) in *CALHM1* reduces plasma membrane Ca^2+^ permeability and is associated with an earlier age-at-onset of AD. To investigate the role of *CALHM1* variants in early-onset AD (EOAD), we sequenced all *CALHM1* coding regions in three independent series comprising 284 EOAD patients and 326 controls. Two missense mutations in patients (p.G330D and p.R154H) and one (p.A213T) in a control individual were identified. Calcium imaging analyses revealed that while the mutation found in a control (p.A213T) behaved as wild-type CALHM1 (CALHM1-WT), a complete abolishment of the Ca^2+^ influx was associated with the mutations found in EOAD patients (p.G330D and p.R154H). Notably, the previously reported p. P86L mutation was associated with an intermediate Ca^2+^ influx between the CALHM1-WT and the p.G330D and p.R154H mutations. Since neither expression of wild-type nor mutant CALHM1 affected amyloid ß-peptide (Aß) production or Aß-mediated cellular toxicity, we conclude that rare genetic variants in *CALHM1* lead to Ca^2+^ dysregulation and may contribute to the risk of EOAD through a mechanism independent from the classical Aß cascade.

## Introduction

Key neuronal processes including neurotransmission, synaptic plasticity, learning and memory are regulated by intracellular calcium (Ca^2+^) levels, and alterations in Ca^2+^ dynamics have dramatic consequences in signaling cascades, cytoskeleton modifications, synaptic function, and neuronal survival [1,2]. Since the first formal hypothesis was posed twenty years ago [3], many studies have supported the essential role of Ca^2+^ dysregulation in central pathological processes related to Alzheimer’s disease (AD) [4,5]. Disturbances in Ca^2+^ signaling have been evidenced during the initial phases of AD, even before the accumulation of the amyloid ß-peptide (Aß), a pathological hallmark of AD [6-10]. A large body of work also indicates that mutations in genes unequivocally related to AD, such as the presenilins, the amyloid precursor protein or the apolipoprotein-E lead to alterations in Ca^2+^ signaling, which in turn leads to apoptosis, impairment of synaptic plasticity and neurodegeneration [11-16].

Recently, a novel Ca^2+^ conducting channel, named CALHM1 (for *calcium homeostasis* modulator *1*), has been proposed as a key modulator of intracellular Ca^2+^ homeostasis [17-19]. Characterization of this protein disclosed a multipass, highly conserved transmembrane glycoprotein preferentially expressed in the brain. Electrophysiological data have demonstrated that CALHM1 is the pore subunit of a plasma membrane non-selective channel that regulates cortical neuronal excitability and Ca^2+^ homeostasis in response to extracellular Ca^2+^ levels and voltage [20]. Genetic epidemiological studies have suggested that a proline-to-leucine polymorphism (rs2986017) at residue 86 (p. P86L) in *CALHM1* could modify the age-at-onset of AD [17,21-23], albeit this was not observed in all association studies [24,25].

Prompted by the large body of literature relating Ca^2+^ dysregulation to AD, and the possible influence of *CALHM1* gene variation on AD age-at-onset, we investigated if rare variants in *CALHM1* found in early-onset forms of AD (EOAD) could influence Ca^2+^ homeostasis. 

## Materials and Methods

### Patients and controls

Three independent EOAD series from Spain, Sweden and Finland were included in this study. All the patients were previously screened for mutations in Mendelian AD genes, including *PSEN1*, *PSEN2* and *APP*, and found to be negative. The Spanish series consisted of 98 unrelated EOAD patients with a mean age-at-onset of 54.1±5.6 years (range 33-64 years). All patients were selected from a previous multi-center study [26]. One hundred forty-three cognitively healthy Spanish control individuals with an average age at blood extraction of 69.6±13.2 years were recruited from Hospital Sant Pau (Barcelona) and Hospital La Paz (Madrid). An additional 384 neurologically healthy individuals (81±7.6 years) from Hospital Marqués de Valdecillas, were included as a confirmatory group. The Finnish series consisted of 116 EOAD cases (59.7±4.8 years of age at onset, range 43-65 years) obtained from the Kuopio University Hospital, Finland. The Swedish series comprised 70 EOAD patients with a mean age-at-onset of 54.7±5.1 years (range 41-60 years), and 166 controls (75.3±6.1 years) recruited from the Memory Disorder Unit at the Uppsala University Hospital. For all patients, the diagnosis was established according to the National Institute on Neurological Disorders and Stroke, and the Alzheimer's Disease and Related Disorders Association (NINDS-ADRDA) guidelines [27].

### Ethics Statement

Approval was obtained from the ethics committee or institutional review board of each institution responsible for the ascertainment and collection of samples (Hospital Sant, Pau, Hospital Marqués de Valdecillas, Hospital La Paz, Kuopio University Hospital, and Uppsala University Hospital). Written informed consent was obtained from all participants or representatives.

### Genetic analysis

*CALHM1* exons and intronic flanking regions were amplified by PCR using the primers 5'-CTCTGAGCTCTGTTGGTCCC-3' and 5'-AGGCCCCATTTTGAGAGGTAG-3' for exon 1, and primers 5'-CTGGCATAGAGAGTGCTTTGG-3' and 5'-AGTACTGCCCAGCACTGAAAC-3' for exon 2, and Roche FastStart PCR Master Mix polymerase (Roche Diagnostics Corp., Indianapolis, USA). Cycle sequencing was performed on purified PCR products with Applied Biosystems BigDye terminator v3.1 sequencing chemistry and run on an ABI 3100 (Applied Biosystems, California, USA) genetic analyzer as per the manufacturer’s instructions. The sequences were analyzed with Sequencher software, version 4.2 (Gene Codes Corp., Michigan, USA). The p.G330D genotyping was performed by restriction fragment length polymorphism approach using the BtsCI restriction enzyme to digest a PCR-amplified product of 672 base-pairs (bp). DNA from the patient with the p.G330D mutation was used as positive control in all experiments. All fragments were resolved by electrophoresis on 2% agarose gels stained with ethidium bromide. Presence of the mutation resulted in three bands of 271, 250, and 151 bp whereas its absence resulted in two bands of 271 and 401 bp (primer sequences provided upon request). Protein sequences were aligned with ClustalW2 (http://www.ebi.ac.uk/Tools/clustalw2/index.html). Bioinformatic tools PMut [28], Panther version 7 [29] and PolyPhen-2 [30] were used to predict the impact of rare amino acid exchanges on protein structure and function.

### *CALHM1* subcloning and mutagenesis

Human *CALHM1* cDNA was obtained from Origene (Maryland, USA). CALHM1-P86L, CALHM1-R154H, CALHM1-A213T, and CALHM1-G330D non-synonymous changes were introduced with the QuickChange II site-directed mutagenesis kit (Stratagene), and confirmed by direct sequencing.

To detect transfected CALHM1 using confocal microscopy, plasmids containing CALHM1-WT and CALHM1-P86L tagged at the C-terminal with a myc tag as previously described [17] was kindly provided by Philippe Marambaud. Plasmids containing CALHM1-R154H, CALHM1-A213T, and CALHM1-G330D were introduced with the QuickChange II site-directed mutagenesis kit (Stratagene), and confirmed by direct sequencing.

### Cell culture and transfections

HEK 293 cells, HEK cells stably expressing the Swedish APP mutation (HEK-APPsw, generous gift from Dennis Selkoe) [31], and CHO cells expressing wild-type APP and wild-type PSEN1 (Ps70) [32], were used in this study. Cells were cultured in DMEM with 10% fetal bovine serum (FBS) at 37°C with 5% CO_2_ in a tissue culture incubator. All transfections were performed using Fugene (Invitrogen, California, USA), polyethylenimine ExGen500 (Fermentas MBI) or XtremeGene (Roche) according to manufacturer’s instructions. Conditioned medium was collected 6-24 h after transfection. To compare the expression of the transfected wild-type and mutant CALHM1, Ps70 cells were transfected with CALHM1 tagged with myc. Twenty four hours after transfection, the cells were fixed with 4% paraformaledhide and immunostained for transfected CALHM1 using mouse anti-myc (9E10, 1:100, EMD Millipore Corporation, USA) and goat anti-mouse Alexa555 antibodies (1:200 Life Technologies Corporation). Confocal images were taken with an SP5 confocal microscope (Leica Microsystems GmbH, Wetzlar, Germany) using a 555nm wavelength (63x objective; 4x zoom). Confocal images were taken in multiple z planes (1micron apart) in order to capture the whole cell. The z plane at the midpoint of the cell was used for the confocal images presented here. Confocal images (Figure S1) showed that transfected mutant and wild-type CALHM1 were equally and highly expressed in Ps70 cells and demonstrated a similar pattern of cellular localization.

### Ca^2+^add-back assay

Transfected cells were grown in normal DMEM (containing 1.8 mM Ca^2+^) plus 10% FBS. Twenty-four hours after transfection cells were washed three times with CaCl_2_/MgCl_2_-free Hanks’ balanced salt solution (HBSS), supplemented with 1.7 mM MgCl_2_ and then incubated for 30 min in this medium. Ca^2+^-free HBSS was removed and cells were maintained for 6 hours in HBSS with 1% FBS. Cells were then washed with phosphate-buffered saline (PBS) and conditioned medium was collected for Aβ measurements.

### Calcium imaging

Cytosolic Ca^2+^ signals were obtained from cells loaded with 4.5 μM fura-2 AM as previously described [33]. Cytosolic [Ca^2+^] alterations are presented as the ratio of emitted fluorescence (510 nm) after excitation at 340 nm and 380 nm, relative to the ratio measured prior to cell stimulation (ratio 340/380). Imaging experiments were conducted in an isotonic bath solution (10 mM Tris-HEPES, pH 7.3, 140 mM NaCl, 2.5 mM KCl, 1.2 mM CaCl_2_, 0.5 mM MgCl_2_, 5 mM glucose). Osmolarity was adjusted to 310 mOsm using mannitol.

### Aβ measurements

Human Aβ was measured by ELISA in the conditioned medium 6 hours after Ca^2+^ add-back. The levels of Aß_1-40_ and Aß_1-42_ were measured using commercial enzyme-linked immunosorbent assay kits (IBL, Gumna, Japan) as previously described [34].

### Aβ_1-42_ oligomer preparation

Synthetic Aβ_1-42_ (EZBiolab) oligomers were obtained by dissolving 300 μg freeze-dried aliquots in 20 μL DMSO. Peptide stock aliquots were diluted in 0.1 M Tris-HCl at pH 7.4 to a final concentration of 88.6 μM Aß. Solutions were stirred continuously at 37°C and 300 rpm for 3 h and kept at -80 °C before being used.

### Cell viability assay

HEK cells over-expressing CALMH1-WT, CALHM1-P86L and CALHM1-G330D were seeded in 96-well plates at a density of 3x10^4^ cells/100μL/well. Then 2 µM Aβ_1-42_ oligomers were added for 24h. Cell viability was measured by 3-

(4,5-dimethylthiazol-2-yl)-2,5-diphenyltetrazolium bromide (MTT) reduction as previously described [35]. Briefly, 11 μL of MTT stock solution (5 mg/mL) were added and after 2 h the reaction was stopped with 120 μL of DMSO. MTT reduction was determined in a plate reader spectrophotometer at 540 and 650 nm. The values for untreated cells were taken as 100%.

### Statistical analysis

Data are expressed as mean±SEM for every set of experiments. Statistical analysis was assessed with one-way analysis of variance (ANOVA) using Sigma-Plot and GraphPad Prism 5 software.

## Results

### Identification of *CALHM1* mutations

**Figure 1:**
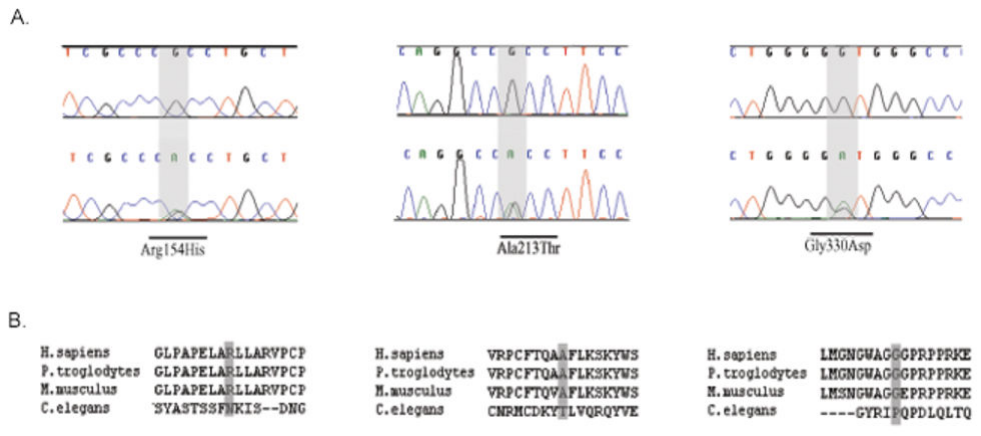
Novel non-synonymous mutations identified in the *CALHM1* gene. **A**. Electropherograms showing wild-type (upper) and mutated (bottom) sequences with the *CALHM1* missense mutations highlighted. **B**. Partial protein sequence alignment of CALHM1 from various species. The sequence of human CALHM1 (NM_001001412.3) was aligned with its orthologs in *Pan* *troglodytes* (XM_521596.2), *Mus* *musculus* (NM_001081271.1) and *Caenorhabditis* *elegans* (NM_063002.2).

Mutation screening of the *CALHM1* gene in EOAD patients disclosed a heterozygous G to A transition (c.G989A), which results in the replacement of a glycine to aspartic acid at codon 330 (p.G330D) in a Spanish patient. In addition, a p.R154H missense mutation (c.G461A) was found in a Swedish patient (Figure 1A). Exhaustive *CALHM1* genetic screening in the control series disclosed just one rare amino acid change (p.A213T), which was found in a healthy individual from the Swedish series (Figure 1A). To rule out that the p.G330D was a common variant within the Spanish population, 384 healthy additional controls belonging to the same geographic ancestry were subjected to RFLP analysis. The genetic variant was not found in any of these control samples.

The patient carrying the p.G330D mutation was a female in whom the first clinical symptoms appeared at the age of 56. Her father had been diagnosed with AD at the age of 65 and died at the age of 68 years. Also, a 78 year old first-cousin has been suffering from dementia since the age of 60. Neither DNA nor further detailed clinical information was available from these two affected family members. The patient with the p.R154H mutation was a female in whom symptoms started at the age of 55 and who died at the age of 76. Both her mother and maternal uncle had dementia at the age of 60 years. The analysis of the only available DNA sample from this family, belonging to a 63 year-old healthy daughter, did not disclose this genetic variant.

The potential pathogenicity of these three missense mutations was assessed *in silico* by three different softwares (Table 1). Both PMut and Panther predicted profound perturbations of protein function in mutations carried by EOAD patients (p.R154H and p.G330D), whereas no pathological effects were anticipated for the p.A213T variant carried by a control individual. Predictions from the PolyPhen-2 software indicated a damaging effect for the p.R154H variant but also for the p.A213T, whereas no deleterious effects were predicted for the p.G330D variant. All substitutions are conserved across vertebrate species (Figure 1B).

**Table 1:** Predictions of the functional impact of novel CALHM1 variants.

	Mutation	PMut*	Panther†	PolyPhen-2‡
Spanish series				
EOAD (n=98)	p.G330D	Pathological (0.64)	Pathological (-3.21)	Benign (0.069)
Controls (n=160)	-			
Swedish series				
EOAD (n=70)	p.R154H	Pathological (0.58)	Pathological (-4.15)	Probably damaging (0.993)
Controls (n=166)	p.A213T	Neutral (0.14)	Not pathological (-2.23)	Probably damaging (0.995)
Finnish series				
EOAD (n=116)	-			

^*^Prediction of pathological consequences of the amino acid exchange predicted by PMut software. A pathogenicity index ranging from 0 to 1 is provided. Mutations associated with an index above 0.5 are considered pathological.

^†^Panther classification system of each of the amino acid substitutions. The numbers in parenthesis refer to the substitution position-specific evolutionary conservation score (based on an alignment of evolutionarily related proteins), from 0 (neutral) to about - 10 (most likely to be deleterious). - 3 is the previously identified cut-off point for functional significance.

^‡^PolyPhen-2 refers to the pathogenicity prediction on Polymorphism Phenotyping, version 2. Prediction scores are presented in parenthesis (zero indicates benign and 1 damaging).

### *CALHM1* missense mutations alter Ca^2+^ homeostasis

**Figure 2:**
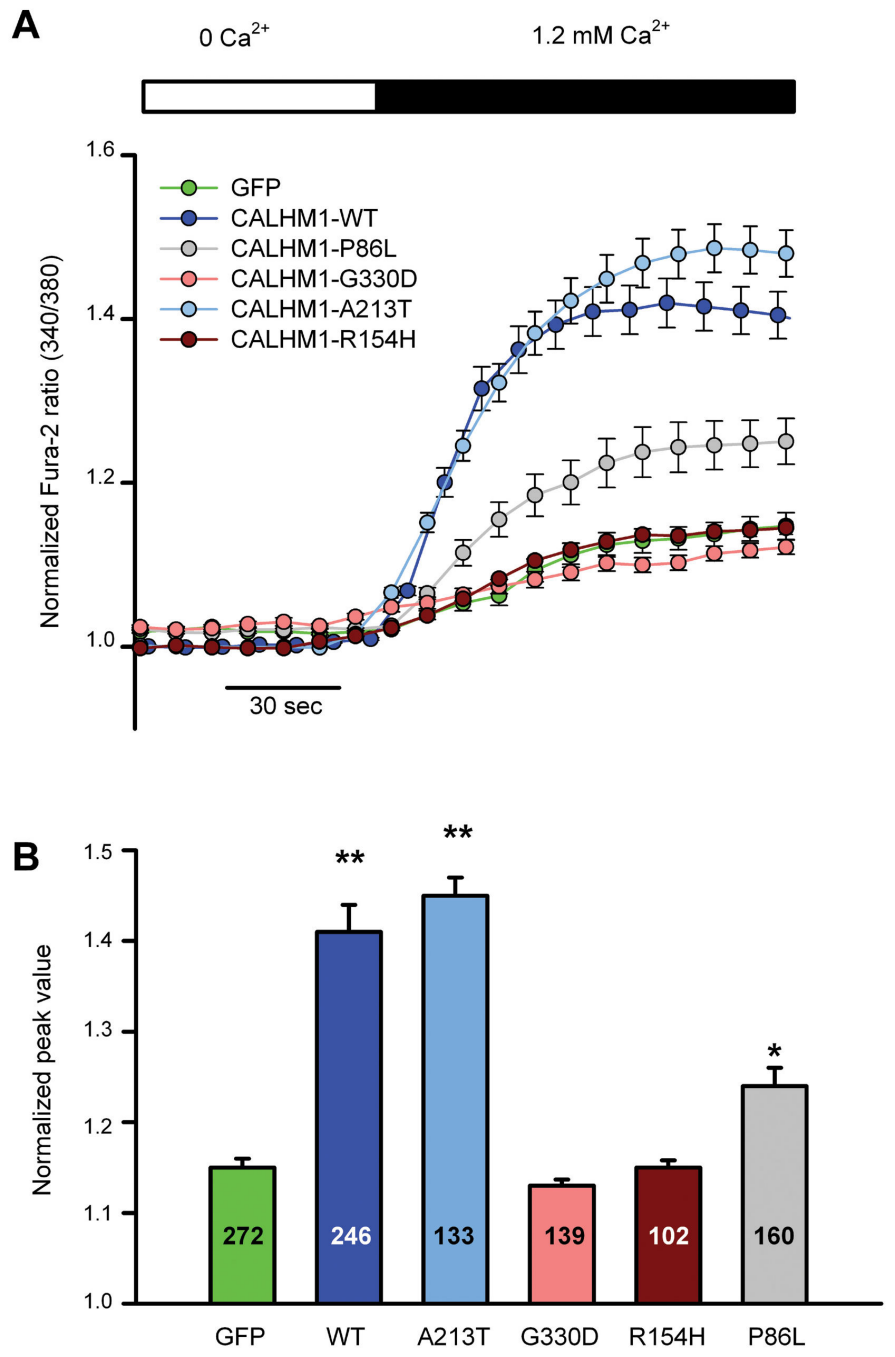
Effect of CALHM1 mutations on Ca^2+^ influx. **A**. HEK-293 cells transfected with GFP, CALHM1-WT, CALHM1-P86L, CALHM1-R154H, CALHM1-A213T and CALHM1-G330D were loaded with Fura-2 and tested for Ca^2+^ influx into the cytoplasm following the removal of Ca^2+^ from the extracellular media. Time course of Ca^2+^ signals normalized to the value before the addition of Ca^2+^ and expressed as the mean±S.E.M. are shown. **B**. Mean peak Ca^2+^ signals for the different conditions are shown. Number of cells analysed are given for each condition. * *P*<0.05 *vs* GFP; ** *P*<0.01 *vs* GFP, one-way ANOVA and Bonferroni *post* *hoc*.

To address whether these variants affect Ca^2+^ entry into the cytosol, we conducted Ca^2+^ influx measurements in HEK-293 cells expressing wild-type or mutant CALHM1 and loaded with the cytosolic Ca^2+^ dye Fura-2 (Figure 2A). Mean increases in the peak Ca^2+^ signal measured 1 min after the addition of Ca^2+^ to the extracellular solution are shown in Figure 2B. Following a transient depletion of extracellular Ca^2+^, and consistent with previous reports, we observed that re-addition of extracellular Ca^2+^ to cells transfected with CALHM1-WT generated a strong and sustained increase in cytosolic Ca^2+^ concentration. This intracellular Ca^2+^ elevation was significantly higher than in cells expressing the CALHM1-P86L polymorphism, also in accordance with a previous report [17]. As shown in Figure 2B, the two mutations identified in EOAD patients, *CALHM1*-G330D and *CALHM1*-R154H, produced further attenuated Ca^2+^ increases that were no different from control GFP-transfected cells. On the other hand, the CALHM1-A213T mutation found in a healthy subject generated a Ca^2+^ influx similar to CALHM1-WT.

### *CALHM1* mutations do not alter Aβ levels or Aβ-mediated cell toxicity

**Figure 3:**
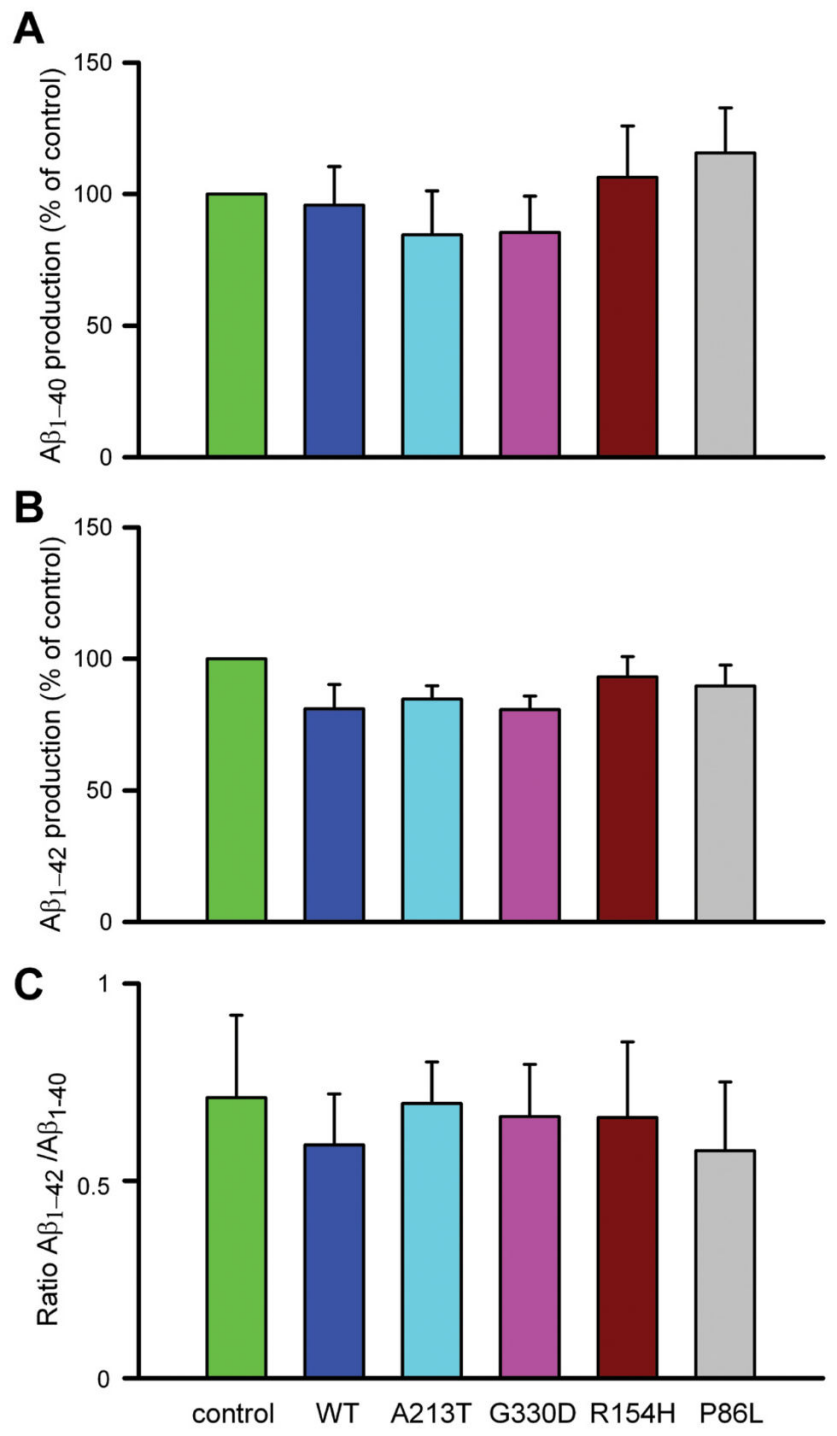
Effect of CALHM1 mutations on the production of Aβ_**1-40**_ **and** Aβ_1-42_. HEK- APPsw cells transfected with GFP and the different CALHM1 constructs were tested for the production of (**A**) Aβ_1-40_ and (**B**) Aβ_1-42_. The ratio Aβ _1-42_/Aβ_1-40_ is shown in C. Data are mean±SEM values of four independent experiments.

We next determined whether these novel CALHM1 variants influence Aβ levels. We transfected HEK-APPsw cells with wild-type and mutant CALHM1 constructs and measured Aβ levels by ELISA in the medium 6 and 24 hours after Ca^2+^-add-back. We did not find any clear effect of these variants on Aβ40 (Figure 3A) or Aβ42 levels (Figure 3B), and the Aβ42/Aβ40 ratios were similar in all cases (Figure 3C). Similar results were obtained in CHO cells co-expressing APP-WT and CALHM1 proteins (data not shown).

**Figure 4:**
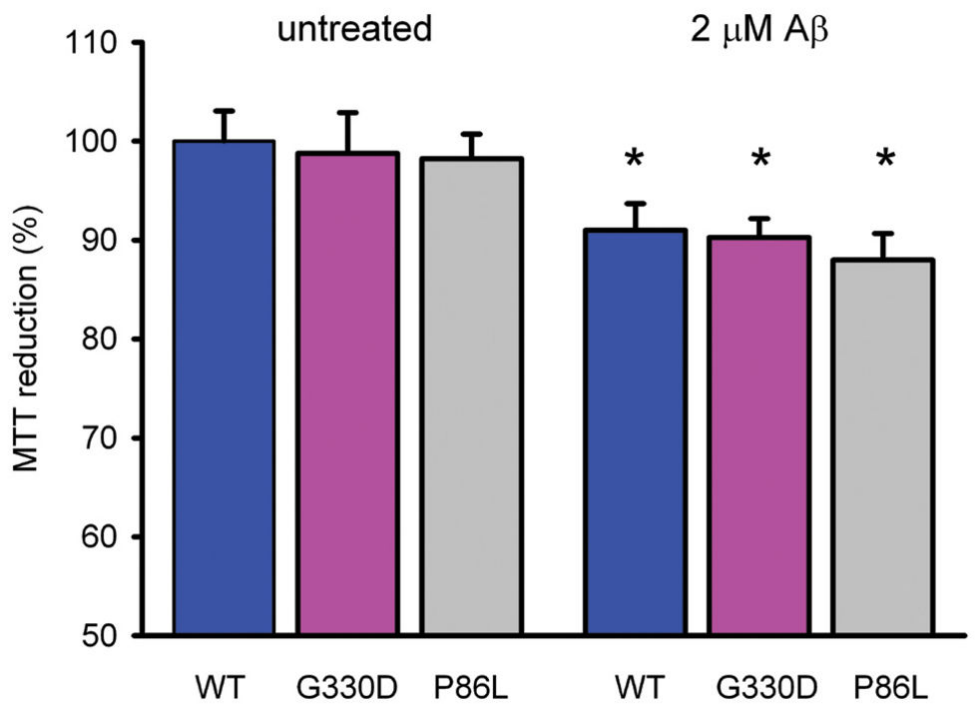
Effect of CALHM1 on **Aβ**_1-42-_ induced cell toxicity. HEK-293 cells over-expressing CALHM1-WT, CALHM1-G330D and CALHM1-P86L were treated with 2 μM Aβ_1-42_ oligomers for 24h. Cell viability was measured by MTT reduction. Data are the mean ± SEM of three independent experiments performed in triplicate. * *P*<0.05 vs untreated cells by student t test.

We also tested whether the expression of different CALHM1 proteins modified cell viability in the absence or the presence of 2 μM Aβ oligomers. Cell viability was measured by MTT reduction in HEK-293 cells transiently transfected with CALHM1-WT, CALHM1-P86L and CALHM1-G330D (Figure 4). Cell viability was equally reduced in the presence of Aβ for both wild-type and mutant CALHM1-expressing cells, therefore discarding a role of disease associated mutations of CALHM1 in Aβ-induced cell toxicity. Similar results were obtained when cells over-expressing the different CALHM1 proteins were challenged with hydrogen peroxide (data not shown).

## Discussion

Our systematic mutation screening of *CALHM1* in patients with EOAD has revealed two novel missense variants (p.R154H and p.G330D) in two patients and a non-synonymous variant (p.A213T) in a control individual. *In silico* analyses by two independent methods (PMut and Panther) predicted that the p.R154H and p.G330D missense variants, but not p.A213T, would have important effects on protein function. However, a third predictor (PolyPhen-2) yielded inconclusive results. We therefore performed a comprehensive analysis of these three rare genetic variants to assess their influence on the functional properties of the CALHM1 ion channel. Our results are intriguing because they strongly indicate that the p.R154H and p.G330D genetic variants found in EOAD patients with a positive family history of AD have important consequences in CALHM1 function (namely, extracellular Ca^2+^ influx), whereas the p.A213T variant carried by an asymptomatic elderly control did not. Furthermore, we found that the p. P86L polymorphism, previously shown to modify the age-at-onset of AD, had an intermediate effect between CALHM1-WT and the novel pathogenic variants. In contrast to a previous report [17], we could not find any changes in extracellular Aß levels or Aß-mediated toxicity in cells expressing either the p. P86L or any of the variants described here, despite their more severe effect on CALHM1 channel activity. Our data strongly argue against a direct impact of CALHM1 on Aβ production or Aß-mediated cell toxicity. Instead, we postulate that the implication of CALHM1 in the pathophysiology of AD is most likely related to CALHM1-induced Ca^2+^ dyshomeostasis. In fact, disruption of Ca^2+^ signaling is an early event in the pathogeneis of AD, prior to cognitive decline and neuronal death, and expression of genes associated with Ca^2+^signaling have been shown to be significantly altered in concomitance with the progression of Alzheimer’s pathology [10]. Since intracellular Ca^2+^ levels are tightly controlled in cells, these sustained disturbances in Ca^2+^ homeostasis lead to a wide range of harmful processes such as synaptic transmission alterations and plasticity impairment [5]. Therefore, although the exact mechanism underlying CALHM1-induced changes in the pathogenesis of AD cannot be elucidated from our data, we hypothesize that alterations in CALHM1 properties can have significant consequences in early AD pathways, including neuronal excitability [20], and synaptic function [36-38].

Taken together, our results suggest that rare genetic variants in CALHM1 disturb Ca^2+^ homeostasis and may contribute to the development of EOAD. More generally, our data strongly support the calcium hypothesis as a relevant event in the pathogenesis of AD. We propose that the mechanism by which CALHM1 dysregulation may contribute to AD is unlikely related to Aβ production and cytotoxicity, and that a potential role of CALHM1 in neuronal excitability merits further investigation.

## Supporting Information

Figure S1 **Wild-type and mutant CALHM1 exhibit similar expression and localization patterns in Ps70 cells.** CALHM1 was visualized using antibodies against the myc tag.
(TIF)
