# Supplementary figures and images for: Rare Variants in Calcium Homeostasis Modulator 1 (CALHM1) Found in Early Onset Alzheimer’s Disease Patients Alter Calcium Homeostasis
Source: PLoS One. 2013 Sep 17;8(9):e74203. doi: 10.1371/journal.pone.0074203 (PMC3775809; doi:10.1371/journal.pone.0074203)

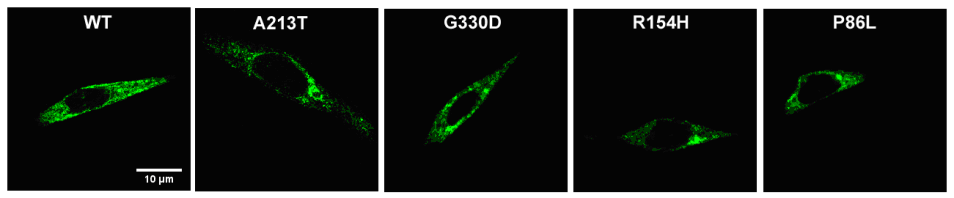

Supplement: Figure S1 — Wild-type and mutant CALHM1 exhibit similar expression and localization patterns in Ps70 cells. CALHM1 was visualized using antibodies against the myc tag. (TIF) [file pone.0074203.s001.tif]
